# Analytical prediction of the piezoelectric *d*_33_ response of fluoropolymer arrays with tubular air channels

**DOI:** 10.1038/s41598-018-22918-1

**Published:** 2018-03-15

**Authors:** Sergey Zhukov, Dagmar Eder-Goy, Sergey Fedosov, Bai-Xiang Xu, Heinz von Seggern

**Affiliations:** 1 Institut für Materialwissenschaft, Technische Universität Darmstadt, Alarich-Weiss-Straße 2, Darmstadt, 64287 Germany; 2 Institut für Materialwissenschaft, Technische Universität Darmstadt, Otto-Berndt-Straße 3, Darmstadt, 64287 Germany; 3 Department of Physics and Materials Science, Odessa National Academy of Food Technologies, ul. Kanatnaya 112, Odessa, 65039 Ukraine

## Abstract

The present study is focused on tubular multi-channel arrays composed of commercial fluoropolymer (FEP) tubes with different wall thickness. After proper charging in a high electric field, such tubular structures exhibit a large piezoelectric ${{\boldsymbol{d}}}_{{\bf{33}}}$ coefficient significantly exceeding the values of classical polymer ferroelectrics and being even comparable to conventional lead-free piezoceramics. The quasistatic piezoelectric ${{\boldsymbol{d}}}_{{\bf{33}}}$ coefficient was theoretically derived and its upper limits were evaluated considering charging and mechanical properties of the arrays. In order to optimize the ${{\boldsymbol{d}}}_{{\bf{33}}}$ coefficient the remanent polarization and the mechanical properties were taken into account, both being strongly dependent on the air channel geometry as well as on the wall thickness of the FEP tubes. The model predictions are compared with experimental *d*_33_ coefficients for two particular arrays with equal air gaps of 250 μm, but with different wall thickness of utilized FEP tubes of 50 μm and 120 μm, respectively. Analytical modeling allows for the prediction that arrays made of FEP tubes with a wall thickness of 10 μm are foreseen to exhibit a superb piezoelectric response of up to 600 pC/N if the height of stadium-like shaped air channels is reduced down to 50 μm, making them potentially interesting for application as highly sensitive sensors and energy harvesting.

## Introduction

New polymer materials called ferroelectrets or piezoelectrets, with internally charged air voids possess high piezoelectric activity and has gained interest in the scientific community in recent years^1,2^. Being initially completely non-polar, ferroelectrets exhibit a strong piezoelectric effect only after symmetry breaking during poling in high electric fields due to positive and negative charge separation in microplasma discharges (the onset of which is governed by Paschen’s law) and subsequent trapping at the polymer/air interface^2–4^. Piezoelectricity originates from thereby engineered dipoles/polarization in the air-filled voids by forming layers with opposite surface charges $\pm {\sigma }_{int}$ at the polymer/air interfaces, which can be considered as creating a polarization ${P}_{int}$. Applying an electric field of opposite sign leads to the polarization reversal like in real ferroelectric materials. The corresponding switching process is described by a hysteresis loop^2,5^ regarded as a typical property of ferroelectrics^6,7^. Moreover, like all piezoelectrically active materials, ferroelectrets also belong to a class of smart materials that allow converting electrical energy into mechanical one and vice versa^6–8^. Since the initially very promising films of cellular polypropylene (PP) ferroelectrets were not sufficiently stable at temperatures above +60 °C^9^, the development of new thermally stable polymers with voids^10–18^ and hybrid structures with artificial air cavities^19–24^ is still ongoing.

**Figure 1:**
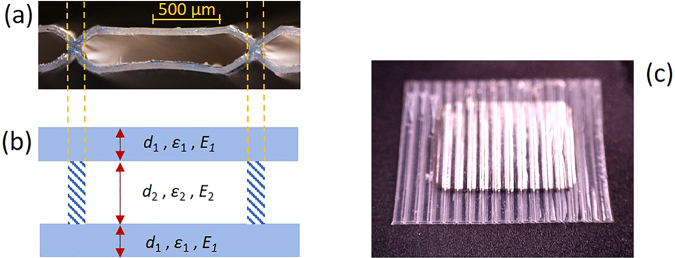
(**a**) A cross section micrograph of fragments of resulting array fabricated at +270 °C from FEP tubes with wall thickness of 50 µm, (**b**) Schematic representation of the tubular array with regular plane-parallel air channels. The thickness of the walls is greatly exaggerated for clarity. (**c**) A photograph of the array with sputtered electrodes.

One of the promising structures is a fluoropolymer array with tubular air channels^25–30^. The tubular channels may be introduced either artificially using a template-based lamination technique^25,26^ or be directly formed from fluorinated ethylene propylene (FEP) tubes^30^. In the latter approach, a set of individual FEP tubes is compressed between two heated metal plates. Under this condition, squeezed FEP tubes are welded together at +270 °C, resulting in a flat array with regular distributed tubular air channels, as shown in Fig. 1(a). The array fabricated from FEP tubes with 1 mm diameter and a wall thickness of 50 µm has a stable and high piezoelectric ${d}_{33}$ coefficient in the range from 120 pC/N to 160 pC/N with a flat frequency response between 0.1 Hz and 10 kHz^30^. However, the tubular array produced from tubes with the thicker wall of 120 µm shows a more than twofold decrease in the piezoelectric response because of the increased stiffness. Rather high ${d}_{33}$ coefficients from 70 pC/N up to 350 pC/N were reported for tubular structures obtained by template-based lamination, and several approaches for increasing the charging efficiency of the structure were also discussed^25–28^. All above mentioned reports have shown that ferroelectrets composed of tubular air channels are very promising for piezoelectric applications. However, a theoretical estimation of the potential limits of such arrays and ways for optimizing the tubular structure have not been studied so far.

The present paper is an attempt to theoretically analyse the quasistatic piezoelectric ${d}_{33}\,\,$coefficient in dependence on the main array parameters, such as wall thickness and air channel height. For this purpose, previously developed models for cellular polymers^31–35^, as well as for sandwiched arrangements^36–39^ and for hybrid structures^21,24^ have been adapted for the used multi-channel structures. The validity of the proposed model is verified by comparing theoretical and experimental data for the interfacial charge densities and accompanying quasistatic ${d}_{33}$ coefficients. The paper is organized as follows: First, a theoretical model for obtaining the ${d}_{33}$ coefficients is introduced. Second, it is shown how the accumulated interface charge and effective stiffness of the tubular arrays can be derived from the experimental hysteresis loop and measurements of the mechanical response, respectively. Third, theoretical predictions for the piezoelectric ${d}_{33}$ coefficient are compared with the experimental results and fourth, the proposed model is used to estimate ${d}_{33}$ coefficients for hypothetical tubular devices in order to predict the maximum ${d}_{33}$ value and related parameters. Finally, the sample preparation and description of the experimental methods are presented.

## Results and Discussion

### A theoretical model for the piezoelectric ${{\boldsymbol{d}}}_{{\bf{33}}}$ coefficient

The longitudinal piezoelectric ${d}_{33}$ coefficient is used to characterize the piezoelectric response when the external force $F$ is applied in the direction perpendicular to the plane of the array. It is expected that the resulting piezoelectric ${d}_{33}$ coefficient of the array shown in Fig. 1(a) is determined by the utilized geometric, dielectric and mechanical parameters of the material used and also strongly depends on the amount of stored charge at the wall/air interfaces introduced by poling^31–39^. To simplify the model, the tubular structure depicted in Fig. 1(a) is represented by two planar FEP films separated by a gap as shown in Fig. 1(b). In order to consider the influence of the necking of the structure indicated by the area between the two dotted lines in Fig. 1(a) and (b), a correction factor $\alpha$ is introduced. We note that an analogous approximation for the tubular structure was used in refs^22,25^. For the upcoming calculations it is also assumed that the electric field strengths are constant in the air-filled channel as well as in the walls. In other words, there are only charges existing at the interfaces between air and FEP^31^. Another assumption is that the elastic deformation of the array obeys Hooke’s law with a constant Young’s modulus. Additionally, the wall thickness ${d}_{1}$ is assumed to remain constant, and only the air-filled channel ${d}_{2}$ is deformed under the external stress. Under such conditions, the mechanical stress ${\sigma }_{mech}$ can be defined as:

1 $${\sigma }_{mech}=\frac{F}{A}={Y}_{total}\cdot \,[\frac{\Delta {d}_{2}}{2{d}_{1}+{d}_{2}}],$$

where $F$ is the externally applied force to the tubular structure with the sample area $A$, ${Y}_{total}$ the Young’s modulus of the whole device, and ${\rm{\Delta }}{d}_{2}$ the thickness change of the air layer under the applied force $F$.

The piezocoefficient ${d}_{33}$ is defined as:

2 $${d}_{33}=\frac{\Delta Q}{F}=\frac{\Delta Q/A}{F/A}=\frac{{\varepsilon }_{0}{\varepsilon }_{1}\Delta {E}_{1}}{{Y}_{total}\cdot [\frac{\Delta {d}_{2}}{2{d}_{1}+{d}_{2}}]},$$

where $\Delta Q$ is the measured change of the electrode charge, and $\Delta {E}_{1}$ the electric field change in the FEP wall adjacent to the electrode caused by the external mechanical force $F$.

Under the assumption of a sandwich consisting of one air layer of thickness ${d}_{2}$ and two equally thick solid FEP layers of thickness ${d}_{1}$ without edge effects, the electric fields ${E}_{1}$ and ${E}_{2}$ in the FEP layers and in the air gap, respectively, are obtained by using Gauss’ law and Kirchhoff’s second law under short-circuit conditions as follows:

3 $${\varepsilon }_{0}{\varepsilon }_{1}{E}_{1}={\varepsilon }_{0}{\varepsilon }_{2}{E}_{2}+{\sigma }_{int},$$

4 $$2{d}_{1}{E}_{1}+{d}_{2}{E}_{2}=0,$$

where ${\sigma }_{int}$ are the trapped areal charge density at the air/FEP interfaces. Under these conditions one obtains for the electric field ${E}_{1}$ in the solid walls from Equations (3) and (4):

5 $${E}_{1}=\frac{{\sigma }_{int}}{{\varepsilon }_{0}{\varepsilon }_{1}\,+\,{\varepsilon }_{0}{\varepsilon }_{2}\,\frac{2{d}_{1}}{{d}_{2}}\,}.$$

The expression ${\rm{\Delta }}{E}_{1}/{\rm{\Delta }}{d}_{2}$ as needed in Equation (2) is obtained by differentiation of Equation (5):

6 $$\frac{\Delta {E}_{1}}{\Delta {d}_{2}}=\frac{{\varepsilon }_{0}{\varepsilon }_{2}\,\frac{2{d}_{1}}{{d}_{2}^{2}}{\sigma }_{int}}{{({\varepsilon }_{0}{\varepsilon }_{1}+{\varepsilon }_{0}{\varepsilon }_{2}\frac{2{d}_{1}}{{d}_{2}})}^{2}}.$$

Consequently, the ${d}_{33}$ piezocoefficient of a planar structure without edges becomes:

7 $${d}_{33}\,=\alpha \cdot \frac{{\varepsilon }_{1}{\varepsilon }_{2}\,{\sigma }_{int}}{{Y}_{total}}\cdot \frac{1+({d}_{2}/2{d}_{1})\,}{{({\varepsilon }_{2}+{\varepsilon }_{1}({d}_{2}/2{d}_{1}))}^{2}}.$$

where *α* is introduced as a correction factor taking care of the differences between the idealized sandwich structure neglecting edge effects and the experimental sample structure as shown in Fig. 1(a) and (b). The fasciated area in Fig. 1(b) resembles thereby the deviating structure from the experimental sample of Fig. 1(a) which is responsible for the correction factor $\alpha$. Equation (7) without the correction factor can also be deduced from ref.^31^ modeling the piezoelectric response of cellular PP by a larger number of layers. It is obvious that ${\sigma }_{int}$ and ${Y}_{total}$ have to be optimized to obtain the highest possible piezoelectric activity.

How this can be achieved and how the deviations from the idealized structure assumed for Equation (7) can be taken into account will briefly be reviewed. First, the interface charge density ${\sigma }_{int}$ has to be optimized. For a structure displayed in Fig. 1(b), there is no piezoelectric activity in the areas without air gap connecting adjacent channels as marked by the dashed lines. Therefore, the above introduced correction factor $\alpha$ is needed to describe the air-filled array to the total area ratio of the array^24^. Below it will be shown how $\alpha$ can be determined experimentally. According to theoretical models describing similar devices^5,36–38^ with two solid blocking layers separated by an air gap, an increase of the poling voltage *V* across the entire structure results in the increase of an electric field ${E}_{2}$ in the air gap and ${E}_{1}$ in the walls as shown in Fig. 1(b). When ${E}_{2}$ reaches the threshold value ${E}_{B}$ for air, breakdown starts in the air channels. This occurs at a surface potential ${V}_{B}$. Taking again into account Equations (3) and (4), the interface charge density dependence on poling voltage $V$ can be solved as^5,36,37^:

8 $${\sigma }_{int}(V)=\{\begin{array}{ll}0 & \,V\le {V}_{B}\\ {\varepsilon }_{0}[\frac{{\varepsilon }_{1}}{2{d}_{1}}V-(\,{\varepsilon }_{2}\,+\,\frac{{\varepsilon }_{1}{d}_{2}}{2{d}_{1}}){E}_{B}] & \,V\ge \,{V}_{B}\end{array},$$

where the threshold voltage ${V}_{B}$ is related to the breakdown field ${E}_{B}$ by:

9 $${V}_{B}=({d}_{2}+\frac{2{\varepsilon }_{2}{d}_{1}}{{\varepsilon }_{1}}){E}_{B}.$$

In Equation (8) for $V > {V}_{B}$ the breakdown field ${E}_{B}$ stays constant in the air channel since a linear increase of voltage will results in a linear increase in ${\sigma }_{int}$ with the slope $\frac{{\varepsilon }_{0}{\varepsilon }_{1}}{2{d}_{1}}$. This slope is determined solely by the wall thickness and its dielectric constant. During the measurement of the hysteresis, the poling voltage changes from $+V$ to $-V$, while the accumulated interface charge ${\sigma }_{int}$ initially remains constant due to stable charge trapping until the electric field in the air channel ${E}_{2}$ resumes the value ${E}_{2}=-{E}_{B}$. At this point, a renewed breakdown allows for the reversal of the interface charge. As a result, ${\sigma }_{int}(V)$ follows a parallelogram-like hysteresis loop during the charging cycle^5,36,37^.

It should be noted that for practical purpose the remanent interface charge ${\sigma }_{rem}$ is important, i.e. the interfacial charge ${\sigma }_{int}$ after an applied voltage has been turned off or the sample was short-circuited. This parameter can be expressed as^5,36,37^:

10 $${\sigma }_{rem}(V)=\{\begin{array}{ll}0 & \,V\le {V}_{B}\\ {\varepsilon }_{0}[\frac{{\varepsilon }_{1}}{2{d}_{1}}V-(\,{\varepsilon }_{2}+\frac{{\varepsilon }_{1}{d}_{2}}{2{d}_{1}}){E}_{B}] & \,{V}_{B}\le V\le 2{V}_{B}\\ ({\varepsilon }_{2}{\varepsilon }_{0}+{\varepsilon }_{1}{\varepsilon }_{0}\frac{{d}_{2}}{2{d}_{1}}){E}_{B}={\sigma }_{rem}^{max} & \,V\ge 2{V}_{B}\end{array}.$$

where ${\sigma }_{rem}^{max}$ is the maximum remanent interface charge obtained during short-circuiting after the poling voltage has been at least $2{V}_{B}$. Further increase of the poling voltage would not result in higher remanent polarization due to the back-switching during short-circuiting the sample^5,37,40^.

It is important to note that Equation (10) indicates that each voided structure has a certain limit for the stored interface charge density. If now Equation (10) is substituted into Equation (7), the corresponding maximum value of the piezoelectric coefficient can be written as:

11 $${d}_{33}^{max}=\alpha \frac{{\varepsilon }_{0}{\varepsilon }_{1}{\varepsilon }_{2}{E}_{B}(2{d}_{1}+{d}_{2})}{{Y}_{total}\cdot (2{\varepsilon }_{2}{d}_{1}+{\varepsilon }_{1}{d}_{2})}$$

It should be noted that Equation (11) is applicable only in the case that the applied poling voltage has reached or exceeded the value of $2{V}_{B}$. For lower poling voltages, Equation (7) should be used.

### Interfacial charge density ${{\boldsymbol{\sigma }}}_{{\boldsymbol{int}}}$ and its hysteresis in tubular-channel ferroelectrets

**Figure 2:**
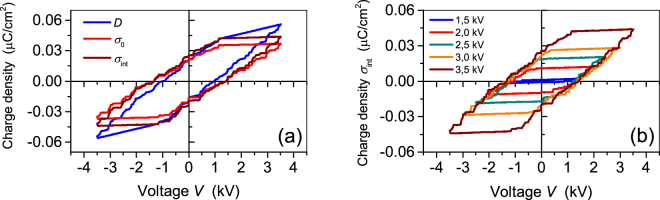
(**a**) Experimentally determined *D* and calculated ${\sigma }_{0}$ and ${\sigma }_{int}$ hysteresis loops at a peak voltage of ±3.5 kV for a 350 µm thick array fabricated from tubes of 50 µm thick walls and having a geometric capacitance *C*_S_ of 12 pF, (**b**) ${\sigma }_{int}$ hysteresis loops recorded for the same specimen for different peak voltages as indicated. The measurements were carried out at a frequency of 1 Hz.

In order to investigate the evolution of the interfacial charge density ${\sigma }_{int}$ on applied voltage, measurements of electrical hysteresis loops of the fabricated arrays were conducted with the Sawyer-Tower circuit^41,42^. To this end, the voltage ${V}_{out}$ built on the large capacitor ${C}_{0}$ in series with the sample was measured by means of a Keithley 2000 multimeter, and the charge flowing through the circuit was determined as:

12 $$Q(t)={C}_{0}{V}_{out}(t)={C}_{S}{V}_{S}(t)+A{\sigma }_{0}(t)$$

where ${V}_{out}(t)$ is the actually measured potential across ${C}_{0}$, $A$ the sample area and ${C}_{S}$ geometric capacitance of the array, while$\,{\sigma }_{0}$ is the electrode charge induced by the interfacial charge ${\sigma }_{int}$ at the wall/air interface as introduced previously. Figure 2(a) displays the measured displacement $D=Q/A$ at an applied peak voltage of ±3.5 kV, and the derived hysteresis loops for ${\sigma }_{0}$ and ${\sigma }_{int}$ for the array composed from 50 µm thick walls.

Hysteresis loops for the charge density ${\sigma }_{0}$ in the metal electrodes are obtained from the measured displacement *D* by subtracting the term ${C}_{S}{V}_{S}(t)$ (see Equation (12)) whereby the geometric capacity *C*_S_ of the arrays was determined independently with an LCR Meter (HP Model 4332 A). For the studied arrays, the geometric capacitance varied from 8 pF to 12 pF due to the utilized wall thickness. As a next step, the interfacial charge density ${\sigma }_{int}$ was calculated from the charge density ${\sigma }_{0}\,\,$by considering the thickness of the air channel ${d}_{2}$ and the wall thickness ${d}_{1}$, and the respective dielectric constants ${\varepsilon }_{1}$ and ${\varepsilon }_{2}$. Then, application of second Kirchhoff’s law (Equation (4)) for short-circuit and Gauss’ law (Equation (3)) yields:

13 $${\sigma }_{0}=\frac{{\varepsilon }_{1}{d}_{2}}{2{\varepsilon }_{2}{d}_{1}+{\varepsilon }_{1}{d}_{2}}{\sigma }_{int}=k\quad {\sigma }_{int},$$

where $k$ is a geometry dependent factor of proportionality. Taking into account that ${\varepsilon }_{1}=2.1$ and ${\varepsilon }_{2}=1$, ${d}_{2}=250\,\mu m$ and ${d}_{1}=50\,\mu m$ (thin-wall array) or 120 µm (thick-wall array), the factor $k$ is 0.84 and 0.69, respectively.

One can also see from Fig. 2(a) that hysteresis loops for ${\sigma }_{0}$ and ${\sigma }_{int}$ exhibit the shape of a tilted parallelogram exactly as predicted by the model described above. A certain deviation of the curves from ideal parallelograms can be caused by deviations of the geometry of the air channels from the assumed rectangular or plane-parallel shape (compare Fig. 1(a) and (b)). Calculated ${\sigma }_{int}$ loops for the same sample but at different peak voltages are displayed in Fig. 2(b). In accordance with Equations (8) and (9), all the loops for the selected tubular array have the same slope and the same width equal to $2{V}_{B}$, but different amplitudes which are proportional to the peak voltage. No hysteresis loop for ${\sigma }_{0}$ and ${\sigma }_{int}$ could be recorded for the peak voltage less than 1.4 kV, which indicates that the breakdown voltage ${V}_{B}$ is equal ${V}_{B}=1.4\,kV$ for the particular array. This experimentally determined value will be used for further estimates, but it is somewhat less than 1.78 kV, calculated from formula (9) using the prediction of the Paschen law^3^ for an air gap of 250 μm. The main reason for this discrepancy is probably due to the fact that the actual thickness of the air gap in the individual channels can vary and deviate to smaller values due to an inwards bending of the horizontal cell walls as can be seen from Fig. 1(a). Consequently, the experimental air breakdown is initialized at a lower voltage than predicted by the model for the nominal air gap thickness. Despite this fact, it can be concluded that the experimental hysteresis loops for arrays with tubular air channels generally follow the model predictions proposed in the above section.

**Figure 3:**
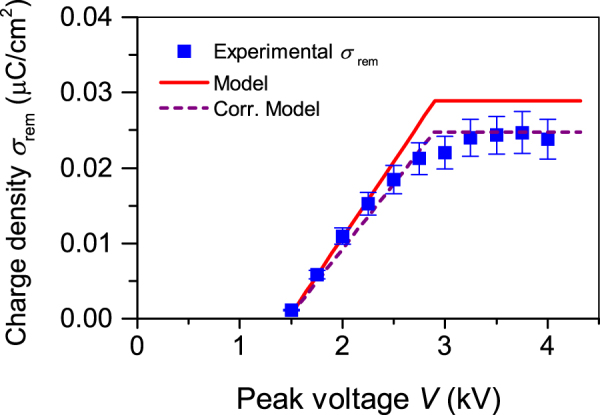
Theoretical (solid line) ${\sigma }_{rem}$ and experimental (symbols) ${\sigma }_{rem}$ versus peak voltage for a 350 µm thick array fabricated from tubes of 50 µm thick walls. The dashed line resembles the corrected charge density of the plane parallel sandwich utilizing $\alpha =0.86$.

In the following, this model will additionally be used to analyse the remanent polarization ${\sigma }_{rem}$ as a function of the peak voltage for the above structure. Corresponding theoretical (see Equation (10)) and experimental results obtained for different peak voltages are depicted in Fig. 3 by the red line and blue squares, respectively. As the model predicted, the theoretically obtained polarization ${\sigma }_{rem}$ increases linearly with increasing peak voltages until it reaches a saturation value ${\sigma }_{rem}^{max}=0.029\,\mu C/{{\rm{cm}}}^{2}$ at a peak voltage of 2.75 kV. The experimentally obtained ${\sigma }_{rem}^{max}$ is smaller and amounts to ${\sigma }_{rem}^{max}=0.025\,{\rm{\mu }}C/{{\rm{cm}}}^{2}$. The difference can be used to determine the above introduced correction factor $\alpha$ as the ratio of the experimental and theoretical charge density, which amounts to $\alpha =0.86$. Another way to look at the correction factor $\alpha$ is to interpret it as the ratio of the array area with air channels to the total area of the array^22,24^. For the array with 50 µm thick walls, such a coefficient can be estimated to $\alpha  \sim 0.90$ (see Fig. 1(b)) which is slightly larger than the previous derivation. The reason can be seen in the rounded ends of the stadium-shaped tubes, whose influence on the deposited charge density and thereby the ${d}_{33}$ coefficient is not yet understood completely. The follow-up investigations in the near future will focus on this issue. It can be seen in Fig. 3 that the corrected model reliably describes the overall behaviour of the remanent charge density ${\sigma }_{rem}$ for different peak voltages.

**Figure 4:**
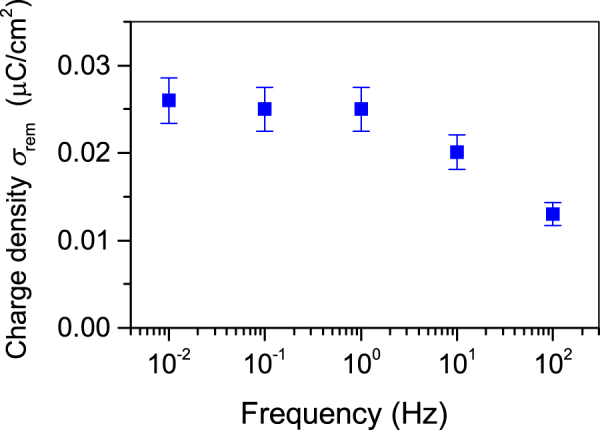
Interfacial charge density ${\sigma }_{rem}$ vs. frequency of the hysteresis cycle for the thin-wall array with air gap of 250 µm.

To estimate the effective switching rate of the polarization in the array, ${\sigma }_{rem}$ was analyzed from the obtained hysteresis loops for peak voltage of ±3.5 kV for different loop cycle frequencies varying from 10 mHz to 100 Hz. Figure 4 displays ${\sigma }_{rem}$ gained from the corresponding cycle frequencies. One realizes that the reversed charge ${\sigma }_{rem}$ is nearly constant up to 1 Hz while at higher frequencies it decreases. Obtained results prove that the polarization switching in the present arrays is a rather fast process. Full reversal can be completed in just one second. This experimental result also indicates that the utilized time of one minute for polarization of virgin samples by the contact method is more than sufficient to reach the maximum polarization or charge density.

**Figure 5:**
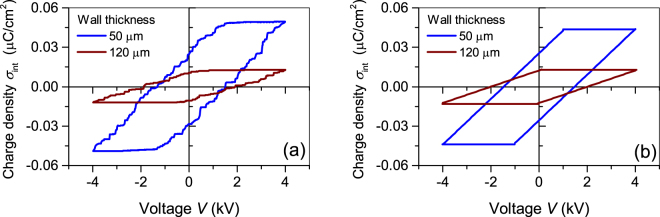
(**a**) Experimental hysteresis ${\sigma }_{int}$ for thin- and thick-wall arrays, as indicated. Both arrays have air channels with the same height of 250 µm. (**b**) Theoretical hysteresis loops calculated from the above theory for the different wall thickness and the respective correction factors $\alpha =0.86\,{\rm{and}}\,\alpha =0.80$.

In order to study the differences in the hysteresis behaviour of thin- and thick-wall arrays the polarization hysteresis has been measured for an equal air channel height of 250 μm utilizing a peak voltage of ±4 kV. The resulting ${\sigma }_{int}$ loops are displayed in Fig. 5(a). As expected from Equation (10), the thicker wall results in a lower slope ${\varepsilon }_{0}{\varepsilon }_{1}/2{d}_{1}$ of the hysteresis loop, thereby reducing ${\sigma }_{rem}^{max}$. Additionally, the use of thicker walls is accompanied by an increase in critical voltage ${V}_{B}$, and thus broadens the loop. It was previously established that the parameter ${E}_{B}$ in Equation (10) should not depend on the wall thickness and therefore is equal for both arrays^36,37^. Like in the case of a thin-wall array, the experimentally determined value of ${V}_{B}\,$= 1.90 kV for the thick-wall specimen is a little bit lower than 2.07 kV, calculated from Equation (9) using the breakdown prediction of Paschen’s law for a corresponding air gap^3^. For a thick-wall array at an elevated peak voltage, the saturation value ${\sigma }_{rem}^{max\,}$ = 0.011 µC/cm^2^ is obtained at a peak voltage of about 4 kV. The model provides the same value for the remanent charge density, if the correction factor of about 0.80 is used for the ratio of the area occupied by the air channels to the total area of the sample.

The ${\sigma }_{int}$ theoretical hysteresis loops for both arrays are displayed in Fig. 5(b). A direct comparison of the experimental and theoretical loops in Fig. 5 reveals that the proposed model for the hysteresis behaviour provides reliable results for the stored interface charge density ${\sigma }_{int}$ for various tubular arrays. For both structures it is also confirmed that the devices have a limit of the remanent charge, which is reached at the poling voltage $V=2{V}_{B}$. The amount of remanent charges is determined by the geometrical and dielectric properties, as well as by the threshold field ${E}_{B}$.

### Mechanical properties of tubular arrays

**Figure 6:**
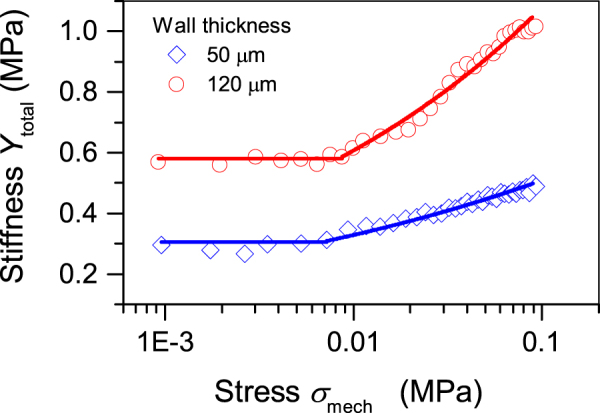
Effective Young’s modulus versus stress for arrays with air gap of 250 µm and different wall thickness as indicated. For ${\sigma }_{mech} > 0.01\,MPa$ solid lines represent fits by the function ${Y}_{total}=a\cdot {\sigma }_{mech}^{n}$, where $a=0.073$ and $n=0.17$ for thin-wall and $a=0.061$ and $n=0.25$ for thick-wall arrays, respectively, while for lower stress the stiffness was kept constant.

Besides the interfacial charge density ${\sigma }_{int}$, Young’s modulus ${Y}_{total}$ of the whole tubular array plays a crucial role for the final piezoelectric ${d}_{33}$ coefficient (see Equations (7) and (11)). To determine the mechanical properties of the two tubular arrays, the quasistatic stress-strain curves were measured at room temperature. Both arrays revealed slightly non-linear mechanical responses for strain levels ${\varepsilon }_{mech}$ below 15%^30^. Figure 6 displays ${Y}_{total}$ determined from the derivative $\partial {\sigma }_{mech}/\partial {\varepsilon }_{mech}\,\,$at different stress levels. As can be seen from Fig. 6, the minimum values for Young’s modulus are about 0.3 MPa and 0.6 MPa for arrays with wall thickness of 50 μm and 120 μm, respectively, indicating that the wall thickness of utilized FEP tubes have a strong impact on the actual stiffness of the fabricated arrays. Moreover, both values remain almost constant for a stress up to about 0.01 MPa, indicating that for such stress level Hooke’s law is valid. For increasing stress, the stiffness for both arrays shows a clear tendency to increase. To obtain a mathematical description of ${Y}_{total}({\sigma }_{mech})$, the experimental results for ${\sigma }_{mech} > 0.01\,MPa$ were fitted by a power function^43^ ${Y}_{total}=a\cdot {{\sigma }_{mech}}^{n}$, whereas for lower stress the stiffness was maintained constant at about 0.3 MPa or 0.6 MPa depending on the wall thickness. The corresponding fits are shown in Fig. 6 as solid lines. It can be seen that such description of ${Y}_{total}({\sigma }_{mech})$ provides an appropriate fit to the experimental results for both arrays.

### The piezoelectric ${{\boldsymbol{d}}}_{{\bf{33}}}$ coefficient: model vs. experiment

**Figure 7:**
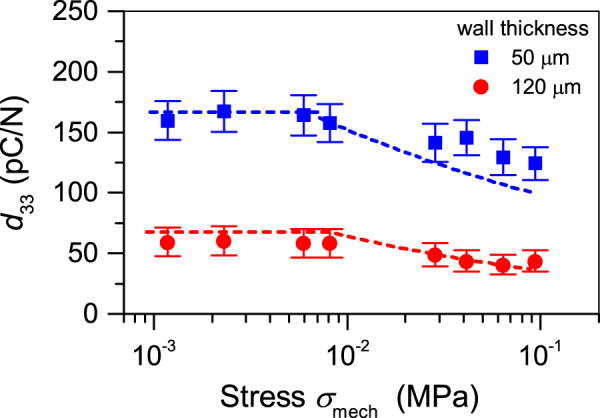
Theoretical (dashed lines) and experimental (closed symbols) piezoelectric ${d}_{33}$ coefficients versus stress for two arrays with air gap of 250 µm and different wall thickness as indicated.

Utilizing the experimentally determined values for ${\sigma }_{rem}^{max}$ and fit function for ${Y}_{total}({\sigma }_{mech})$, the theoretical ${d}_{33}$ coefficient as function of applied stress ${\sigma }_{mech}$ can be calculated utilizing Equation (7). The results of such calculation for two tubular arrays are displayed as dashed lines in Fig. 7. As expected for both arrays, the ${d}_{33}$ coefficient is virtually constant up to 0.01 MPa while at higher applied stress it decreases.

It should be noted that the mechanical compression of the soft tubular channels leads to a redistribution of the electric fields inside the arrays. For an extremely high compression level, it can be expected that the electric field across the air channels may exceed the critical value limited by Paschen’s law^3^. This can cause an unwanted electric breakdown in the air channels, which leads to a partial depolarization of the sample thus lowering ${\sigma }_{rem}$. In turn, this will also lower the piezoelectric response permanently. However, for low compression levels used in the current work, this factor can be neglected. Under this condition, the behaviour of the ${d}_{33}$ coefficient on external stress, shown in Fig. 7, is basically controlled by ${Y}_{total}({\sigma }_{mech})$ as displayed in Fig. 6.

The measured ${d}_{33}$ coefficients vs. stress for both arrays are also shown in Fig. 7. There is a good correspondence between the calculated and measured values. The decrease of the $\,{d}_{33}$ coefficients with stress is explained by the increase of the elastic modulus of the structure due to an obvious densification of the structure at higher stress. Such a behaviour is typical not only for the tubular structures^26^ but also for cellular^43,44^ and open-porous^17,18^ ferroelectrets.

### The piezoelectric ${{\boldsymbol{d}}}_{{\bf{33}}}$ coefficient: arrays with varied *d*_1_ and *d*_2_

**Figure 8:**
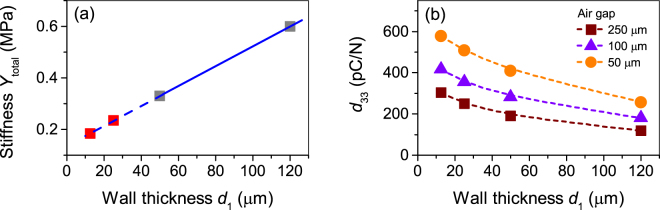
(**a**) Measured (grey squares) and extrapolated (red squares) *Y*_total_ values as a function of wall thickness ${d}_{1}$ for arrays with a common air channel height of ${d}_{2}=250\,\mu m$. (**b**) Theoretical *d*_33_ coefficients for tubular arrays as a function of ${d}_{1}$ for various ${d}_{2}$ values as indicated. Dashed lines are guides to the eye.

To explore future potential of tubular channel arrays, the piezoelectric ${d}_{33}$ coefficient for hypothetical devices with smaller wall thickness and air channel heights will be analysed using Equation (11). Therefore, it is assumed that the sample is poled to its maximal interface charge density ${\sigma }_{rem}^{max}$, while the parameter $\alpha$ is fixed at 0.85. Further simplifications were used to perform such calculations. First, a linear extrapolation for the ${d}_{2}$ dependence of low stress ${Y}_{total}$ for arrays with a common air gap thickness of 250 µm is used on the basis of the wall thickness of 50 μm and 120 μm. The linear extrapolation is shown in Fig. 8(a) and is used to evaluate the stiffness of the samples for ${d}_{1}$ smaller than 50 μm. The values obtained are about 0.25 MPa and 0.20 MPa for the 25 µm and 12.5 µm wall thickness, respectively. Note that FEP solid films with mentioned thickness are available on the market and can in principle be used for the production of such ferroelectrets with tubular channels by template-based lamination^25,26^. It was also assumed that arrays made of tubes with fixed wall thickness, but different air channel heights of 50 μm, 100 μm and 250 μm, virtually have the same Young’s modules. However, it has to be taken into account that the breakdown strength ${E}_{B}$ in Equation (11) depends on ${d}_{2}$. Recent detailed experiments have shown that ${E}_{B}$ in ferroelectrets with air voids with a height of the order of tens of microns exactly follows the prediction of Paschen’s law^45^, while in earlier publications some deviations from this law have been reported^36,37^. For the present estimation the values for ${E}_{B}$ of 110 kV/cm, 86 kV/cm and 60 kV/cm were used for air gaps of 50 μm, 100 μm and 250 μm, respectively.

The estimates of the piezoelectric *d*_33_ coefficients obtained under the above mentioned assumptions for different hypothetical devices with different wall thickness ${d}_{1}$ and air channel heights ${d}_{2}$ are shown in Fig. 8(b). It can be recognized that the theoretical piezoelectric coefficients vary from about 100 pC/N to about 600 pC/N, indicating that the tubular structure can be efficiently optimized to maximize the piezoelectric response. One promising way of such an optimization is to reduce the wall thickness ${d}_{1}$, keeping the thickness of the air channel ${d}_{2}$ constant. Such a modification of the structure is accompanied by a decrease in Young’s modulus, as shown in Fig. 8(a), which leads to a significant increase in ${d}_{33}$. At the same time, the ${E}_{B}$ parameter does not or only slightly depend on ${d}_{1}$, while the term $\frac{2{d}_{1}+{d}_{2}}{2{\varepsilon }_{2}{d}_{1}+{\varepsilon }_{1}{d}_{2}}$ in Equation (11) varies insignificantly. Therefore, mainly the function ${Y}_{total}({d}_{1})$ seems to be dominant for the piezoelectric response of the array when only the wall thickness is changed.

The second possible way to optimize the tubular structure involves regulating the height of the air channel. It can be seen from Fig. 8(b) that a decrease in ${d}_{2}$ from 250 µm to 50 µm enhances ${d}_{33}$ approximately twice. This is valid for all wall thickness used. The main reason for this effect is related to the dependence of ${E}_{B}$ on ${d}_{2}$, since the breakdown field in the air channel increases in accord with Paschen’s law with decreasing ${d}_{2}\,$^3,45^. Once again, we note that possible changes in the stiffness of the array by modification of the air channel height in the current study are not taken into account.

Before concluding, it should be emphasized that the present study has once again demonstrated that the outstanding piezoelectric properties of tubular arrays in particular and ferroelectrets in general originate from a unique combination of mechanical and electrical properties of polymer structures with voids. On the one hand, polymer dielectrics with air cavities can accumulate a quasi-remanent polarization with relatively low charge density up to approximately 0.1 µC/cm^2 ^^22,27,37–39,46^ significantly lower than the typical values for classical bulk ferroelectric polymers (5–15 µC/cm^2^)^7,47^ and conventional piezoceramics (10–100 µC/cm^2^)^6,48,49^. But on the other hand, ferroelectrets, like all porous polymers^1,50^, have an extremely low Young modulus ranging from 0.2 MPa to 10 MPa, depending on the mechanical properties of the polymer matrix and the pore geometry^10,22,30,33,44,51^.

## Conclusion

A theoretical model is proposed for the quasistatic piezoelectric ${d}_{33}$ coefficient of tubular channel arrays. The model takes into account the polarization through interfacial charge densities stored at the wall/air interfaces, as well as mechanical properties through the Young’s modulus of the investigated arrays. Comparison of theoretical results based on model calculations with experimental data contributes to a deeper understanding of involved physics of ferroelectrets with regular tubular air channels. It is shown that the geometry of the produced arrays, mainly the thickness of the wall of the FEP tubes and the air channels, affect the piezoelectric ${d}_{33}$ coefficient. It has been demonstrated that a future increase of ${d}_{33}$ through optimizing the geometry is possible, e.g. by adjusting the thickness of ${d}_{1}$ and ${d}_{2}$. Model predictions suggest that arrays produced by FEP tubes with down to about 10 μm thick walls will exhibit a piezoelectric ${d}_{33}$ coefficient comparable to conventional PZT piezoceramics.

## Methods

In order to fabricate two structures with regular air channels, the fusion bonding technique was used^29^. The arrays were fabricated from FEP tubes with the same outer diameter of 1 mm but with different wall thickness of 50 μm and 120 μm. Both types of FEP tubes were provided by ZEUS Ltd., USA. Figure 1(a) shows a photomicrograph of a cross section of a tubular array composed from tubes with 50 µm thick walls. The dimensions of the manufactured arrays were limited to approximately 30 × 40 mm^2^, due to the fabrication device while the thickness of the air channels in both structures was fixed at 250 μm. The arrays were first metallized on both sides with *Al* electrodes, which have the shape of a square with a side length of 15 mm. A micrograph of the device with sputtered electrodes is shown in Fig. 1(c). Electrical poling was then conducted by a direct-contact charging in ambient air at room temperature by application of a bias voltage of up to ±5 kV from the power supply HSN-35 (FUG GmbH). Typically, one minute was more than enough to fully polarize such arrays.

The quasistatic measurements of the direct piezoelectric ${d}_{33}$ coefficient were performed by rapid loading with a mass *m*. The temporal charge response ${\rm{\Delta }}Q$ of the sample was measured for 10 s by means of a Keithley 610 C electrometer in the charge mode. The ${d}_{33}$ coefficients then were determined from the relation ${d}_{33}={\rm{\Delta }}Q/(mg)$, where $g$ denotes the acceleration of gravity.

The rheometer AR 2000 Ex from TA Instruments was used to determine the mechanical properties. During the measurement, the specimen was compressed between two parallel bars at a rate of 10 μm/s, and the resultant force was measured by a force gauge. Using this technique, quasistatic stress-strain curves were recorded at room temperature which allowed for the determination of Young’s modulus ${Y}_{total}$.

The polarization hysteresis loops of ferroelectrets were obtained by utilizing the Sawyer-Tower circuit where a large standard capacitor ${C}_{0}$ (455 nF) was connected in series with the sample. An *AC* triangular voltage with a frequency between 10 mHz and 100 Hz was applied by a high voltage amplifier (Trek, Model 20/20 C) controlled by an arbitrary waveform generator. All the experiments were carried out at room temperature.
